# Pharmacoepidemiology of Drug Exposure in Intubated and Non-Intubated Preterm Infants With Severe Bronchopulmonary Dysplasia

**DOI:** 10.3389/fphar.2021.695270

**Published:** 2021-07-20

**Authors:** T. Lewis, W. Truog, L. Nelin, N. Napolitano, R. L. McKinney

**Affiliations:** ^1^ Children’s Mercy Hospital, Kansas City, MO, United States; ^2^ Nationwide Children’s Hospital, Columbus, OH, United States; ^3^ Children’s Hospital of Philadelphia, Philadelphia, PA, United States; ^4^ Department of Pediatrics, Division of Pediatric Critical Care Medicine, The Warren Alpert Medical School of Brown University, Providence, RI, United States

**Keywords:** neonate, bronchopulmonary dysplasia, epidemiology - descriptive, diuretic, inhaled steroid, bronchodilator

## Abstract

**Background:** Infants with severe bronchopulmonary dysplasia (BPD) are commonly treated with off-label drugs due to lack of approved therapies. To prioritize drugs for rigorous efficacy and safety testing, it is important to describe exposure patterns in this population.

**Objective:** Our objective was to compare rates of drug exposure between preterm infants with severe bronchopulmonary dysplasia based on respiratory support status at or beyond 36 weeks post-menstrual age.

**Methods:** A cross-sectional cohort study was performed on October 29, 2019. Preterm infants with severe BPD were eligible and details of respiratory support and drug therapy were recorded. Wilcoxon paired signed rank test was used to compare continuous variables between the invasive and non-invasive groups. Fisher’s exact test was used to compare binary variables by respiratory support status.

**Results:** 187 infants were eligible for the study at 16 sites. Diuretics were the drug class that most subjects were receiving on the day of study comprising 54% of the entire cohort, followed by inhaled steroids (47%) and short-acting bronchodilators (42%). Infants who were invasively ventilated (verses on non-invasive support) were significantly more likely to be receiving diuretics (*p* 0.013), short-acting bronchodilators (*p* < 0.01), long-acting bronchodilators (*p* < 0.01), systemic steroids (*p* < 0.01), systemic pulmonary hypertension drugs (*p* < 0.01), and inhaled nitric oxide (*p* < 0.01).

**Conclusion:** Infant with severe BPD, especially those who remain on invasive ventilation at 36 weeks, are routinely exposed to multiple drug classes despite insufficient pharmacokinetic, safety, and efficacy evaluations. This study helps prioritize sub-populations, drugs and drug classes for future study.

## Introduction

Bronchopulmonary dysplasia (BPD) is a chronic lung disease resulting from prematurity and is made worse by mechanical ventilation as well as infectious and inflammatory insults to the developing lungs in the post-natal period. While the pathogenesis of BPD is complex and multi-factorial, there are salient clinical features in established BPD that prompt trials of drug therapy in the inpatient and outpatient setting (Thebaud et al., 2019). These drug classes include corticosteroids to reduce airway and parenchymal inflammation, beta-agonists to dilate constricted airways, diuretics to treat pulmonary edema, and vasodilators to treat pulmonary hypertension. None of the currently used drugs for BPD are FDA approved in this population and few have undergone rigorous efficacy and safety studies in this population (Abman et al., 2017).

There are many efforts underway aimed at improved neonatal and infant therapeutics (Mulugeta et al., 2017). National legislation, new multi-stakeholder collaboratives (International Neonatal Consortium) and pediatric clinical trial networks (PTN, i-ACT) are all working to advance drug approvals. One major issue specific to infants with BPD is extensive off-label drug use and no clear prioritization of which drugs and drug classes should be studied and approved first (Sheehan et al., 2020). Pharmacoepidemiology studies can assess rates of real-world drug exposure in specific patient populations, allowing scientists and regulators to better understand which drugs are used most and may therefore be optimal targets for improved study and labelling.

The goal of this study was to describe the modern-day point prevalence of drug exposure in a cohort of infants with severe BPD (sBPD), as defined by the 2001 consensus recommendation from the National Institutes of Health workshop: receipt of supplemental oxygen for >28 days in infants born <32 weeks gestation with a need for ≥ 30% fraction of inspired oxygen (FiO_2_) and/or positive pressure respiratory support (nasal continuous positive airway pressure CPAP (CPAP), or invasive mechanical ventilation) at 36 weeks post-menstrual age (PMA) (Jobe and Bancalari, 2001). In addition, we are interested in understanding variability in drug exposures by respiratory support status.

## Methods

The BPD Collaborative designed and executed a multi-site point prevalence study on mechanical ventilation use in this population. In addition, for this study, all centers provided data on drugs that the de-identified study subjects were receiving. All participating sites received institutional review board approval prior to data submission.

This is a cross-sectional cohort study performed on a single day across multiple academic centers which are all part of the BPD Collaborative. The study took place on October 29, 2019. On that day, eligible infants were less than 24 months of age, were <32 weeks gestational age (GA) at birth and had a PMA ≥ 36 weeks on the study day. Eligible infants received at least 28 days of O_2_ after birth and were currently on invasive mechanical ventilation or non-invasive support with ≥ 30% O_2_ or nasal CPAP or high flow nasal canula (HFNC) >2 L per minute. Infants were excluded if they were deemed by local study staff to be on non-invasive support or mechanical ventilation for surgical, neurologic, or anatomic (i.e., congenital lung malformations, airway anomalies, etc.) reasons. These infants were excluded because BPD was not the reason they were receiving respiratory support. For data analysis, the cohort was divided into two binary groups, invasive mechanical ventilation (*via* a tracheostomy or endotracheal tube) versus non-invasive respiratory support (everyone else).

The study created a comprehensive list of drugs used in the treatment of sBPD (furosemide, bumetanide, hydrochlorothiazide, spironolactone, albuterol, levalbuterol, atrovent, formoterol, fluticasone, budesonide, beclamethasone, hydrocortisone, dexamethasone, prednisone, prednisolone, methylprednisolone, ranitidine, pantoprazole, omeprazole, sildenafil, bosentan, inhaled nitric oxide, Treprostinil, iloprost, epoprostenol). Using this list, the study team at each site recorded drug exposure on the study day for eligible infants. To accomplish data abstraction, local study members opened the central RedCap database and examined each infant’s local electronic medical record. Data from the electronic medical record was transferred to the research RedCap database. Only standing medication orders (not PRN or one-time orders) were included in the dataset. Data was abstracted from the chart at 8 am local time on the day of the study.

Based on rates of drug exposure in infants with severe BPD (Bamat et al., 2019), we estimated that 80% of intubated infants and 50% of non-intubated infants would be on the most used BPD drug class, diuretics. Using this predicted different in exposure, enrolling 87 infants gives an 80% power to detect a difference in groups using a *p*-value of 0.05 with two-tailed testing. Wilcoxon paired signed rank test was used to compare continuous variables between the invasive and non-invasive groups. Fisher’s exact test was used to compare binary variables by respiratory support status (invasive versus non-invasive).

## Results

A total of 16 sites from 15 centers across the United States submitted data. The number of infants from each site that met inclusion criteria on the study day ranged from 3 to 28 infants. A total of 187 of the 192 infants had sufficiently complete ventilation and drug information data for data analysis.

Infants who remained on invasive mechanical ventilation at 36 weeks PMA had a median birth weight of 636 g (IQR 540,889), median gestational age at birth of 25 weeks (IQR 24,27) and median PMA of 52 weeks (IQR 44,64) on the study day. Infant who were on non-invasive respiratory support on the study day had a median birth weight of 750 g (IQR 625,950; *p* = 0.009 compared to invasive mechanical ventilation), median gestational age at birth of 25 weeks (IQR 24,27; *p* = 0.91) and median PMA of 43 weeks (IQR 40,47; *p* < 0.0001) on the study day (Table 1).

**TABLE 1:** Entire cohort and ventilation group demographics.

	Entire cohort (*N* = 187)	Invasive ventilation (*N* = 94)	Non-invasive ventilation (*N* = 93)	*p*-value^a^
Gestational age at birth (binned by week)^b^	25 (24,27)	25 (24,27)	25 (24,27)	0.91
Birthweight (grams)^b^	710 (560,940)	636 (540,889)	750 (625,950)	0.009
Male, N	112	55	57	0.77
Female, N	75	39	36	
PMA on study day (binned by week)^b^	46 (41,57)	52 (44,64)	43 (40,47)	<0.0001
Weight on study day (grams)^b^	4,437 (3100,6085)	5,435 (3780,7606)	3,690 (2838,4775)	<0.0001

a Invasive versus non-invasive ventilation groups.

b Median (IQR).

PMA is post-menstrual age.

**FIGURE 1:**
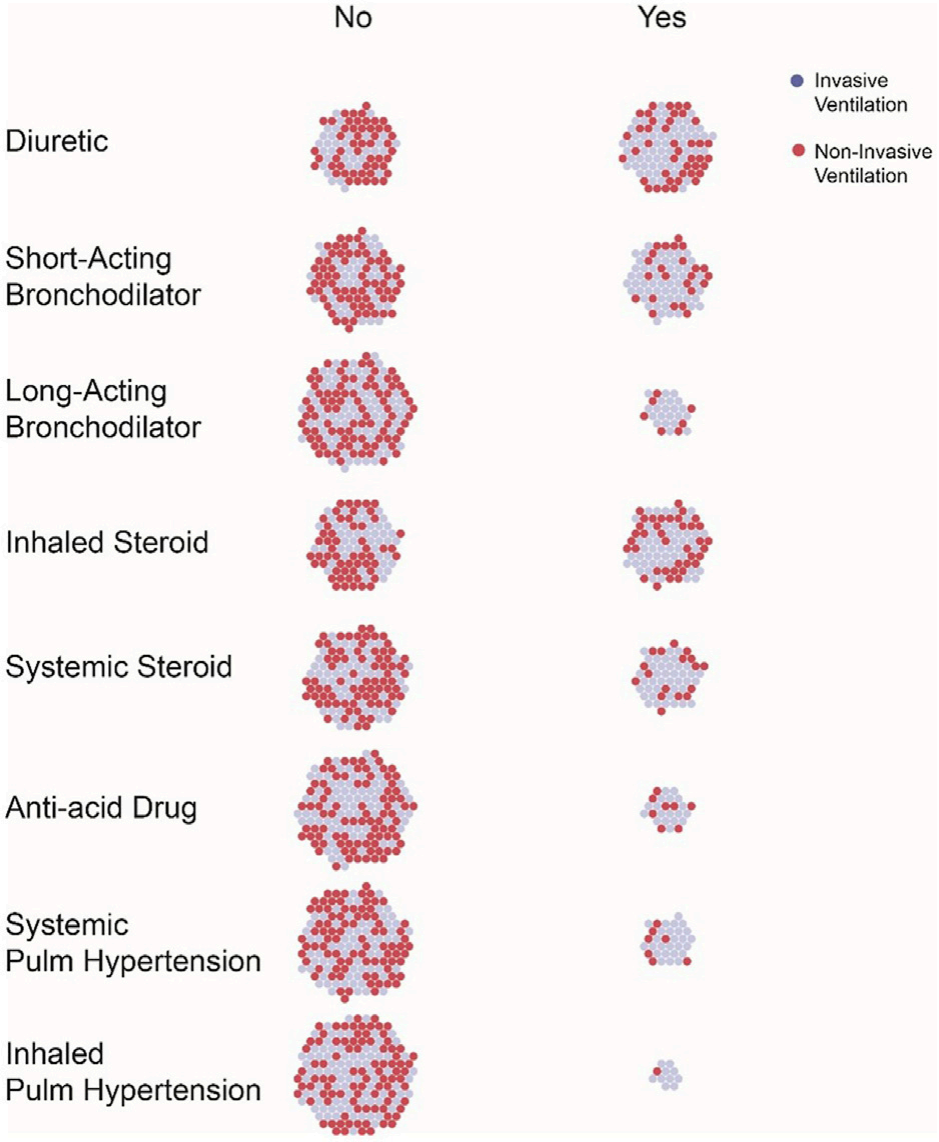
Drug Class Exposure by Ventilation Status.

Point prevalence of drug exposures were assessed by drug class (i.e., Diuretics) and by individual drug exposures (i.e., furosemide). Rates of drug exposure by drug class are presented in Table 2; Figure 1. Diuretics were the drug class that most subjects were receiving on the day of study (54%), followed by inhaled steroids (47%) and short-acting bronchodilators (42%). Inhaled nitric oxide (NO) was the drug that fewest subjects were on the day of the study (6%), followed by anti-acids (14%), and long-acting bronchodilators (15%). Seventeen percent of the entire cohort was on no medications on the study day: 4% in the invasive mechanical ventilation group and 13% in the non-invasive respiratory support group (p = <0.01).

**TABLE 2:** Point Prevalence of drug exposures by ventilation group.

	Invasive ventilation (*N* = 94)	Non-invasive ventilation (*N* = 93)	*p*-value
Drug Class			
Diuretics	60 (64)	42 (45)	**0.013**
Furosemide	36 (38)	19 (20)	**0.01**
Bumetanide	1 (1)	1 (1)	1.00
Hydrochlorothiazide	38 (40)	30 (32)	0.29
Spironolactone	5 (5)	10 (11)	0.19
Short-acting bronchodilator	55 (59)	24 (26)	**<0.01**
Albuterol	54 (57)	21 (23)	**<0.01**
Levalbuterol	2 (2)	3 (3)	0.68
Long-acting bronchodilators	21 (22)	7 (8)	**0.007**
Atrovent	21 (22)	4 (4)	**<0.01**
Formoterol	0 (0)	3 (3)	0.12
Inhaled steroids^a^	49 (52)	38 (41)	0.14
Fluticasone	26 (28)	20 (22)	0.40
Budesonide	24 (26)	18 (19)	0.38
Systemic steroids	40 (43)	17 (18)	**<0.01**
Hydrocortisone	20 (21)	8 (9)	**0.02**
Dexamethasone	7 (7)	4 (4)	0.54
Prednisolone	17 (18)	7 (8)	0.05
Methylprednisolone	2 (2)	0 (0)	0.50
Anti-acid drugs	18 (19)	8 (9)	0.056
Ranitidine	9 (10)	5 (5)	0.41
Pantoprazole	1 (1)	0 (0)	1.00
Omeprazole	8 (9)	3 (3)	0.21
Systemic pulm hypertension	26 (28)	7 (8)	**<0.01**
Sildenafil	26 (28)	6 (6)	**<0.01**
Bosentan	3 (3)	2 (2)	1.0
Inhaled nitric oxide	10 (11)	1 (1)	**<0.01**

Data are displayed as N (%).

Bold *p*-value indicates 2-tailed Fisher’s Exact test < 0.05.

a No infants on the study were treated with beclomethasone.

Infants who were invasively ventilated (verses non-invasively) were significantly more likely to be receiving diuretics (p 0.013), short-acting bronchodilators (*p* < 0.01), long-acting bronchodilators (*p* < 0.01), systemic steroids (*p* < 0.01), systemic pulmonary hypertension drugs (*p* < 0.01) and inhaled nitric oxide (*p* < 0.01). There was no significant difference in exposure to inhaled steroids or anti-acid drugs by ventilation status (Table 2).

## Discussion

In this cohort of infants with severe BPD, over half the infants receiving invasive mechanical ventilation were exposed to daily diuretics, short-acting bronchodilators, and/or inhaled steroids. These common drug exposures occur despite lack of FDA labelling for these medications in this patient population. These data confirm that pediatric providers are using these drugs commonly in real-world settings, and that there is an urgent need for collaboration between providers and regulators to complete the needed safety, efficacy and pharmacokinetic studies to confirm that we are using the right doses in the right infants at the right time.

A very interesting finding of this study is that, in almost all cases, drug exposure is higher in those infants who remain invasively ventilated. This could reflect a phenomenon in which providers do not give drugs to infants who are more “well” and use drugs more frequently in infants with more severe lung disease. On the other hand, the high prevalence of these drug exposures in the children with more severe lung disease, as manifest by their invasive ventilation, may suggest that these drugs are not efficacious in modifying BPD severity or progression, at least at the timing and doses most used. It is also possible that this subset of infants who remain intubated at and after 36 weeks would be even sicker without the use of these drugs. Large, well designed randomized clinical trials are urgently needed to understand the role of these drugs in sBPD.

Some of the most common drug exposures have been associated with drug toxicities. 38% of the infants invasively ventilated were on daily furosemide. This is very different than the standard use of furosemide in the Neonatal ICU, which is a median exposure of 2 days and cumulative exposure of less than 4 mg/kg (Thompson et al., 2020). Furosemide has been associated with secondary hyperparathyroidism in an infant with tracheostomy and severe BPD (Srivastava et al., 2017). Chronic furosemide has also been associated with hearing loss and nephrolithiasis. In a study of a large cohort of preterm infants who received furosemide for more than 28 consecutive days and who were propensity score matched to a non-exposed group of infants (Wang et al., 2018), prolonged furosemide exposure was associated with a trend toward an increase in abnormal hearing screens (Wang et al., 2018). A large systemic review published in 2018 showed no increased risk of sensorineural hearing loss or nephrocalcinosis in preterm infants exposed to furosemide but noted that the quality of evidence was low (Jackson et al., 2018).

The exposure to inhaled medication in over 40% of our cohorts is similar to that found in prior reports of medication use in Neonatal ICU for BPD (Allen et al., 2003; Guaman et al., 2015; Slaughter et al., 2015; Bamat et al., 2019). Most research has involved preterm infants and those with evolving BPD (Wilkie and Bryan, 1987; Rotschild et al., 1989; Pfenninger and Aebi, 1993; Lee et al., 1994; Sivakumar et al., 1999; Morrow et al., 2015; Bassler et al., 2018), however little research has been performed to determine safety and efficacy for infants with sBPD. Napolitano et al., investigated the safety and tolerability of two doses of albuterol (1.25 and 2.5 mg) against placebo (3% saline) in infants with sBPD requiring invasive mechanical ventilation (Napolitano et al., 2021). Infants that responded to albuterol did with the 2.5 mg dose and showed a reduction in PIP while on volume ventilation with no clinically meaningful change in heartrate. There have been no trials on the utilization of inhaled steroids for infants with established sBPD.

Many of the children in this cohort were on systemic steroids, 48% in the invasively ventilated group and 18% in the non-invasive respiratory support group. The most used systemic corticosteroids were hydrocortisone and prednisolone. The finding of hydrocortisone use in this relatively older neonatal population (older than 36 weeks PMA) is unexpected and may reflect prolonged adrenal insufficiency after early-life systemic steroid exposure. Unfortunately, we do not have the data to test this postulate. The second most commonly used steroid, prednisolone, has not been extensively studied in sBPD. In the largest cohort published, 43 infants received prednisolone at an average age of 42 weeks PMA and were treated for a median of 67 days (Linafelter et al., 2019). In this cohort, there was modest short-term improvement in Pulmonary Severity Score at one week with no benefit beyond that, and linear growth was impaired. In our study, 52% of infant receiving invasive mechanical ventilation were on inhaled steroids. While there are published studies on the utility of inhaled steroids to prevent BPD (Shinwell et al., 2016), there is little research on the use of inhaled steroids in infants with established BPD.

Drugs to block gastric acid production were also seen in the invasively ventilated group, with one in five infants exposed. New data highlights the long-term risks of early-life acid suppression including increased risks of infection (Canani et al., 2006; Santos et al., 2019) and later allergies (Trikha et al., 2013; Mitre et al., 2018; Robinson and Camargo, 2018). Because of the common belief that aspirated acid reflux can worsen BPD associated pulmonary hypertension, we assessed the number of infants treated simultaneously with anti-acid drugs and pulmonary hypertension drugs. Eight of the 26 infants treated with anti-acid drugs were on a pulmonary hypertension drug (systemic or inhaled). Given limited data on the benefit of these drugs in BPD, we may be exposing infants to drugs with limited clinical benefit and significant clinical harm.

In summary, all the drug use described in this study is classified as “off-label” meaning there is no FDA labelling to support safety nor efficacy in infants with established BPD. The authors posit that BPD is the quintessential example of a common and severe disease for which no specific therapies have been developed or proven, leaving clinicians to borrow drugs from other patient populations and other diseases and use them to the best of their knowledge. This is clearly unacceptable given the risks of all the drug classes studied and underscores the need to urgently prioritize and perform drug pharmacokinetic, safety, and efficacy trials with a goal of drug labelling in infants with sBPD. When loss of equipoise has occurred, as manifest by common off-label use, this task becomes more challenging. The unique data captured by our study, clearly demonstrates patterns of drug use in sBPD and can inform the design of future studies to enrich for patient populations which are already being clinically exposed to the drug of interest.

## Data Availability

The raw data supporting the conclusions of this article will be made available by the authors, without undue reservation.
